# Stellate ganglion block for preserving arteriovenous fistula in hemodialysis patients undergoing major lower limb orthopedic surgeries: randomized control trial

**DOI:** 10.1186/s12871-025-03150-7

**Published:** 2025-05-31

**Authors:** Ayman Mohamady Eldemrdash, Soudy S. Hammad, Tarek S. Hemaida, Taha Tairy Dardeer, Ahmed Adel Mohsen, Ahmed Khalaf Fathy, Gamal Hendawy Shams

**Affiliations:** 1 Anesthesiology, Surgical Intensive Care and Pain Medicine Department, Faculty of Medicine, Aswan University, Aswan, Egypt; 2 Diagnostic and Interventional Radiology Department, Faculty of Medicine, Aswan University, Aswan, Egypt; 3 Vascular Surgery Department, Faculty of Medicine, Aswan University, Aswan, Egypt; 4 Anesthesiology, Surgical Intensive Care and Pain Medicine Department, Faculty of Medicine, Kafrelsheikh University, Kafrelsheikh, Egypt

**Keywords:** Arteriovenous fistula, Complication, Failure, Hemodialysis, Orthopedic, Stellate ganglion block

## Abstract

**Background:**

Major lower limb orthopedic surgeries can lead to hemodynamic alterations and increase the risk of arteriovenous (AV)fistula thrombosis. This study assessed the role of stellate ganglion block (SGB) in preserving the AV fistulas in hemodialysis (HD)patients undergoing major lower limb orthopedic surgeries.

**Methods:**

In this randomized, controlled, double-blind trial, 50 chronic renal failure patients (ASA physical status III, aged 21–75 years) scheduled for major lower limb orthopedic surgeries were randomized into two groups: Group S received an ultrasound-guided SGB before spinal anesthesia, while Group C received a sham procedure. AVF function was assessed using Doppler ultrasonography on postoperative days 1 and 7. Primary outcome was AVF flow rate. Secondary outcomes included peak systolic velocity (PSV), end-diastolic velocity (EDV), resistive index (RI), thrombosis rate, and functional failure.

**Results:**

Group S demonstrated significantly higher AVF flow rates on both postoperative day 1 (276.96 ± 49.66 ml/min vs. 217.44 ± 46.73 ml/min) and day 7 (254.96 ± 49.38 ml/min vs. 204.56 ± 47.11 ml/min), with large effect sizes (Cohen’s d = 1.23 and 1.04, respectively; *p* < 0.001). PSV and EDV were significantly improved, and RI was significantly lower in Group S. Thrombosis (8% vs. 36%) and failure rates (32% vs. 64%) were significantly reduced compared to the control group (*p* < 0.05).

**Conclusions:**

Pre-emptive stellate ganglion block was associated with significantly improved AVF flow rate postoperatively and reduced thrombosis and functional failure, suggesting its clinical benefit in maintaining AVF patency during major surgeries in HD patients.

**Trial registration:**

This study was approved by the Ethical Committee of Aswan University Hospitals, Egypt (Institutional Review Board (IRB 900/2/24)) and registered on clinicaltrials.gov (ID: NCT06300658). The registration time of this experiment is 3/09/2024. The study protocol was designed and implemented in accordance with the CONSORT guidelines. The study protocol was conducted in compliance with the relevant guidelines and standards.

## Introduction

In patients with chronic renal failure (CRF), hemodialysis (HD) via arteriovenous fistula (AVF) is considered the most effective option, taking into account feasibility, infection rate, and patency [1]. The radio-cephalic fistula between the radial artery and adjacent vein is the most common site for fistula placement [2].

AVF patency is influenced by a range of factors, including both technical and patient-related variables [3], with the internal diameter of the radial artery and its flow being significant predictors [4].

Altered calcium and phosphorus metabolism in CRF patients can lead to increased vascular reactivity [5], making the radial artery more susceptible to calcification and vasospasm compared to healthy individuals. This increased vascular reactivity may contribute to early AVF closure [6]. Several studies have identified various factors that can lead to AVF closure, including inadequate maturation [7], venous stenosis [8], thrombosis [9], and underlying peripheral arterial disease [10].

Hypotension has been identified as a major cause of AVF closure in end-stage renal disease patients [11, 12]. Stellate ganglion blockade (SGB) has been employed for several years in the diagnosis and treatment of circulatory problems in the upper extremity [13]. By blocking sympathetic fibers, SGB reduces vasospasm and improves arterial blood flow. In HD patients undergoing orthopedic surgery, these effects may help maintain AVF patency by preventing intraoperative and postoperative vascular compromise [14].

SGB can inhibit or reduce the muscular layer’s reactivity in the radial artery. This reactivity is triggered by surgical manipulation during the artery’s harvesting and the release of powerful vasoconstrictor mediators during surgery [15].

This study aimed to evaluate the effect of ultrasound-guided SGB on AVF patency and hemodynamic parameters in HD patients undergoing major lower limb orthopedic surgeries.

## Patients and methods

### Clinical trial registration

This study was approved by Aswan university Institutional Review Board (IRB 900/2/24) and written informed consent was obtained from all subjects participating in the trial. The trial was registered prior to patient enrollment at *clinicaltrials.gov* (NCT06300658), Principal investigator: Ayman Mohamady Eldemrdash, Date of registration: 03/09/2024). The enrollment was from March 10, 2024, to July 29, 2024.The study protocol was designed and implemented in accordance with the CONSORT guidelines. The study protocol was conducted in compliance with the relevant guidelines and standards.

This randomized, controlled, double-blind trial comparing Stellate Ganglion Block (SGB) versus a sham procedure in preserving arteriovenous fistula (AVF)function in hemodialysis patients undergoing major lower limb orthopedic surgeries. This trial was carried out on 50 CRF patients aged from 21 to 75 years old, both sexes, American Society of Anesthesiologists (ASA) physical status III, and undergoing major lower limb orthopedic surgeries. Exclusion criteria included patients with ipsilateral brachial and radial artery stenosis, substance abuse, local anesthetic allergy, cardiovascular and respiratory disorders, psychiatric disorders, coagulopathy, vasoactive medication use, body mass index > 30 kg/m^2^, and smoking.

### Randomization

Randomization assignments were done using computer-generated random numbers into two equal groups, Group S (study group): The patient received preemptive SGB. Group C (sham-controlled group): The sham procedure will mimic all steps of the SGB without the actual administration of the local anesthetic. This approach ensures that both groups undergo a similar experience, maintaining the blinding of the study and Allocation concealment was ensured by sealed opaque and sequentially numbered envelopes that opened by a chief nurse who did not take apart in the study. The patient’s group allocation was revealed by opening the sealed opaque envelope containing their assignment before they entered the operating room.

All patients were subjected to medical and surgical history, clinical examination, and laboratory investigations (complete blood count, prothrombin time, liver and renal functions arterial blood gas (ABG), serum Na and serum K).

An 18G cannula was inserted, and monitoring was established using non-invasive blood pressure, pulse oximetry and ECG.

A 25G Quincke spinal needle was inserted into the L3-4 interspace using a midline approach, 12.5 mg (2.5 ml) of hyperbaric bupivacaine 0.5% with 25 mcg fentanyl was injected slowly without barbotage or aspiration.

### Technique of SGB

During SGB procedures strict aseptic technique was used. It was performed by senior anesthesiologists with a minimum of 5 years’ experience in ultrasound-guided nerve blocks. Emergency airway and resuscitation equipment were available in all cases. Complication protocols were established prior to study initiation, including predefined steps for managing pneumothorax, vascular puncture, or other adverse events.

### Group S (study group): -

SGB was performed under ultrasound guidance (GE Logiq P7 ultrasound system). The patients were in supine position, with their head turned 45° to the left, slightly open mouth to allow optimal visualization of the target area and relaxation of the anterior cervical muscles. Then, the skin disinfection was applied.

A high-frequency linear array probe with a frequency range of 6–13 MHz was employed. The probe’s long axis was aligned with the cricoid cartilage’s plane and angled at 45° to the sagittal plane of the neck. The probe was moved from the medial edge of the sternocleidomastoid muscle to the outside. The ultrasound screen successfully visualized the C6 transverse process (TP) and its anterior and posterior tubercles.

**Fig. 1:**
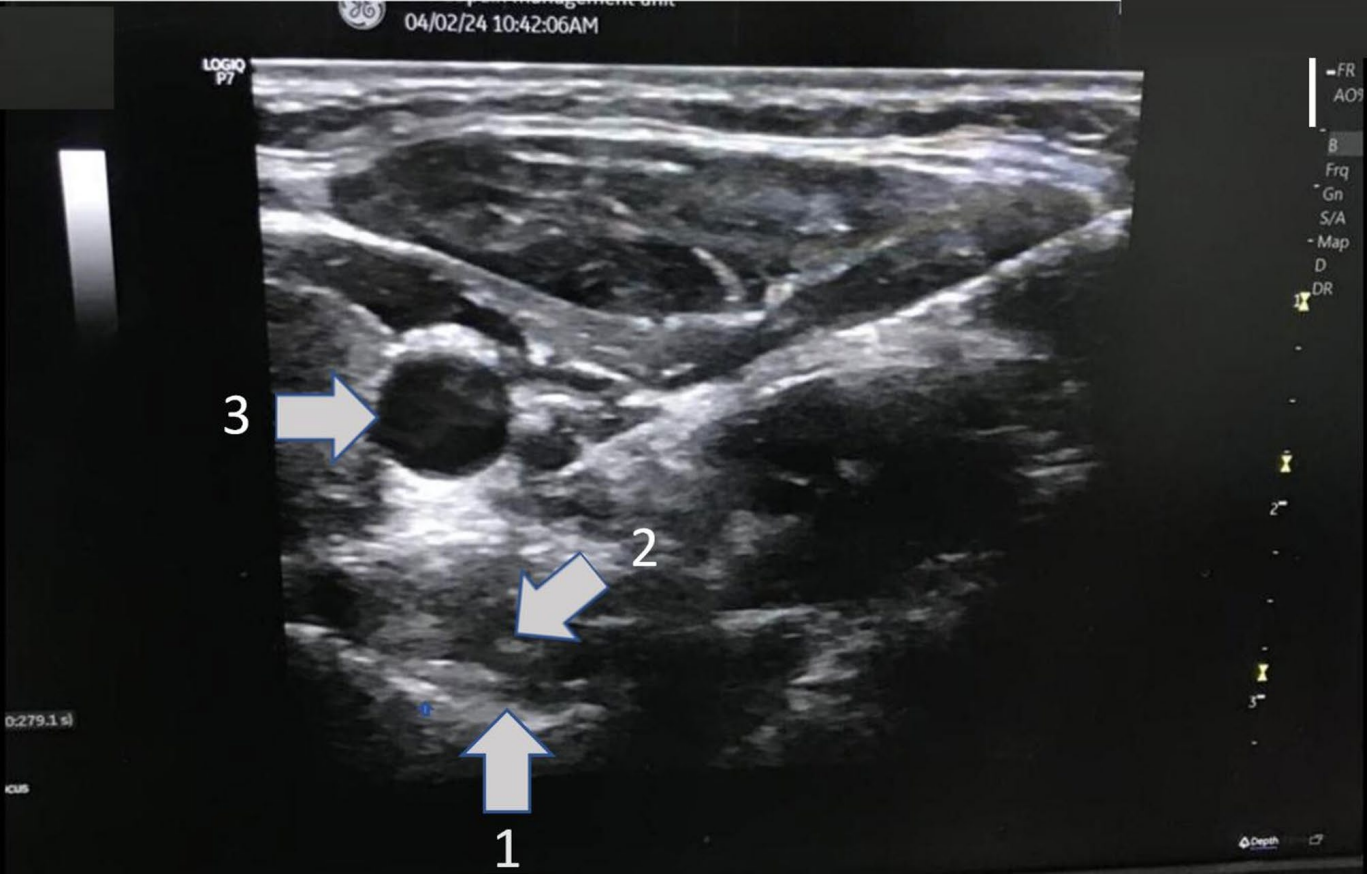
Ultrasonic guided stellate Ganglion block. 1-Transvers process of C6, 2- Longus Colli, 3- Carotid artery

Once the C6 TP was identified, the ultrasound probe was shifted parallel to the tail end until it reached the level of the C7 TP. After carefully examining the anatomical structure, a needle was inserted through the space located on the anterior surface of the longus colli muscle, anterior to the C7 TP, and medial to the common carotid artery and the internal jugular vein. A total of 7.5 ml of bupivacaine 0.25% was injected to complete the block Figure 1.

### Group C (Sham Group)

The sham procedure will mimic all steps of the SGB without the actual administration of the local anesthetic. This approach ensures that both groups undergo a similar experience, maintaining the blinding of the study. The patient will be positioned supine with the neck extended, and the head turned slightly away from the AVF side. Standard monitoring will be in place. Ultrasound will be used to identify the same anatomical landmarks as in the SGB group. The ultrasound probe will be positioned over the transverse process of the C6 vertebra, just as it would be for the actual S group. A small amount of local anesthetic (1–2 mL of lidocaine) will be injected superficially to minimize discomfort at the needle insertion site, as done in the S group. A 22-gauge needle will be inserted to the skin, simulating the process of performing a S group. However, the needle will be deliberately positioned away from the stellate ganglion, and no deep injection will be made. A small amount of saline (e.g., 0.5 mL) will be injected superficially to mimic the sensation of an injection without producing any therapeutic effect. No local anesthetic will be injected near the stellate ganglion. The patient will be observed for the same duration as the SGB group (30 min) and monitored for any immediate reactions.

The volume of blood transfused and the volume of IV fluids infused were both measured. After the operation, the patients were closely monitored for 2 h. Hematocrit (Hct) and Hemoglobin (Hb) levels were measured during this time. Patients were also advised to follow up at the outpatient clinic after 6 weeks and 3 months.

The success of the procedure was determined by the patient’s ability to undergo sufficient HD using the vascular access 6 weeks post-operation for at least 1 month [15].

Doppler ultrasonography was used to measure flow rate of the fistula, PSV, EDV and resistive index (RI).

Adverse effects such as infection, hematoma, bleeding, thrombosis, and ptosis were recorded.

Blood loss was measured according to this modified formula considering IV fluids and blood transfusions were as follows [16]: Blood loss (ml) = EBV × (Initial Hct/Hb − Final Hct/Hb) + volume of transfused blood + volume of IV fluids infused. Where: EBV was the estimated blood volume (calculated based on the patient’s weight). Initial Hct/Hb was the baseline hematocrit or hemoglobin. Final Hct/Hb: was the post-operative hematocrit or hemoglobin. The volumes of transfused blood and IV fluids infused were recorded.

Postoperatively; the flow rate, peak systolic velocity (PSV) and end-diastolic velocity (EDV) were recorded from the radial artery situated two centimeters proximally to the fistula location on both the 1st and 7th days. Additionally, the incidence of failure was documented. Functional failure was defined as the inability to perform effective hemodialysis using the AVF for at least two consecutive sessions within the 6-week follow-up period. Adequate dialysis was defined as achieving a minimum AVF blood flow of ≥ 200 ml/min, as assessed by Doppler ultrasound, and confirmed by nephrology evaluation. The primary outcome was the fistula flow rate. The secondary outcomes were PSV, EDV, RI, incidence of failure, and adverse effects.

### Sample size calculation

The calculation of the sample size was performed using G*Power 3.1.9.2 software (Universitat Kiel, Germany). A pilot study was conducted, involving 10 cases per group, not included in the analysis of the study, which revealed that the mean (± standard deviation) fistula flow rate was 254.10 ± 56.44 in group S and 202.40 ± 62.49 in group C. The determination of the sample size was based on an effect size of 0.868, a 95% confidence level, a study power of 80%, a group ratio of 1:1, and an additional six cases, three for each group to account for potential dropouts. Consequently, a total of 25 patients were recruited for each group.

### Statistical analysis

The statistical analyses were conducted utilizing SPSS version 27 (IBM©, Armonk, NY, USA). The normality of data distribution was evaluated through the implementation of the Shapiro-Wilks test and the examination of histograms. Quantitative parametric data were presented as mean values accompanied by standard deviations (SD) and were analyzed employing the unpaired Student’s t-test. Qualitative variables were reported as frequencies and percentages and were analyzed using the Chi-square test or Fisher’s exact test. A two-tailed P value less than 0.05 was considered statistically significant.

## Results

**Fig. 2:**
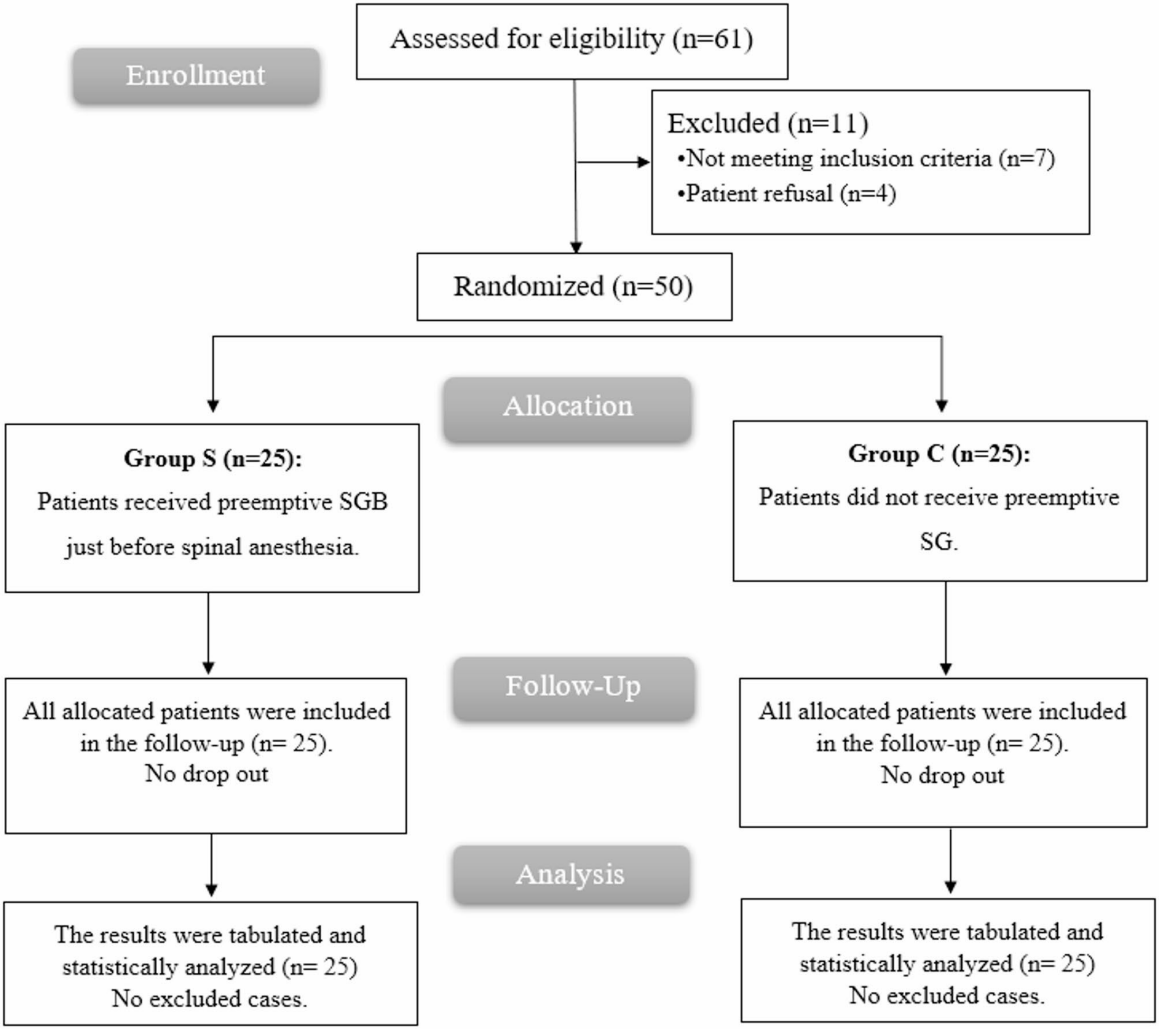
CONSORT flowchart of the enrolled patients

A total of 61 patients were assessed for eligibility; 11 were excluded (7 did not meet inclusion criteria, 4 declined to participate). Fifty patients were randomized into two equal groups (*n* = 25 each) and included in the final analysis (Fig. 2).

Demographic data, comorbidities, fistula site, number of previous AVFs, hematological parameters, transfusion requirements, and intraoperative fluid administration were comparable between groups (Tables 1 and 2).

**Table 1:** Demographic data and fistula characters of the studied groups

	Group S (*n* = 25)	Group C (*n* = 25)	*P* value
Age (years)	50.92 ± 11.41	53.68 ± 12.28	0.414
Sex			0.544
Male	18 (72%)	16 (64%)	
Female	7 (28%)	9 (36%)	
Weight (kg)	70.48 ± 7.79	71.68 ± 6.61	0.560
Height (m)	1.68 ± 0.07	1.66 ± 0.08	0.457
BMI (kg/m^2^)	25.01 ± 2.7	25.94 ± 2.33	0.196
Comorbidities			
DM	9 (36%)	12 (48%)	0.390
Hypertension	12 (48%)	14 (56%)	0.571
Thrill	21 (84%)	18 (72%)	0.306
Site			0.544
Left	18 (72%)	16 (64%)	
Right	7 (28%)	9 (36%)	
Number of previous AVF	2.44 ± 1.08	2.36 ± 0.7	0.758

Data are presented as mean ± SD or frequency (%). BMI: body mass index, DM: Diabetes mellitus. AVF: Arteriovenous fistula

**Table 2:** Hematocrit, hemoglobin, transferred RBCs, volume of IV fluids infused and blood loss of the studied groups

	Group S (*n* = 25)	Group C (*n* = 25)	*P* value
Pre hematocrit (%)	38.84 ± 3.26	39.49 ± 3.16	0.479
Post hematocrit (%)	35.89 ± 3.42	34.27 ± 2.94	0.080
Pre hemoglobin (g/dl)	11.79 ± 1.46	12.23 ± 0.97	0.214
Post hemoglobin (g/dl)	11.29 ± 1.47	10.98 ± 0.94	0.377
Transferred RBCs (units)	2.68 ± 1.11	3.2 ± 1.26	0.127
Volume of IV fluids infused (ml)	292.4 ± 58.54	316 ± 64.42	0.182
Blood loss (ml)	292.4 ± 58.54	317.6 ± 62.93	0.149

Data are presented as mean ± SD. RBCs: Red blood cells. IV: Intravenous

Group S showed significantly higher AVF flow rates than Group C on both postoperative day 1 (276.96 ± 49.66 vs. 217.44 ± 46.73 ml/min; mean difference 59.52 ml/min; *p* < 0.001) and day 7 (254.96 ± 49.38 vs. 204.56 ± 47.11 ml/min; mean difference 50.40 ml/min; *p* < 0.001) (Table 3).

**Table 3:** Peak systolic velocity, end diastolic velocity, fistula flow rate and RI of the studied groups

	Group S *n*= (25)	Group C *n*= (25)	MD and 95% CI	SMD and 95% CI Cohen^’^s d	*P* value
**PSV (cm/sec)**					
1st day postoperative	162.28 ± 35.12	105.84 ± 15.29	(40.85,72.03)56.44	(1.38,2.77)2.08	< 0.001*
7th day postoperative	139.92 ± 35.46	93.84 ± 15.44	(30.33,61.82)46.08	1.68(1.03,2.33)	< 0.001*
**EDV (cm/sec)**					
1st day postoperative	20.52 ± 2.52	17.28 ± 2.05	(1.93,4.54)3.24	1.41(0.78,2.02)	< 0.001*
7th day postoperative	17.64 ± 2.77	13.28 ± 2.19	(2.94,5.77)4.36	1.74(1.08,2.39)	< 0.001*
**Fistula flow rate(ml/min)**					
1st day postoperative	276.96 ± 49.66	217.44 ± 46.73	(32.10,86.93)59.52	1.23(0.62,1.83)	< 0.001*
7th day postoperative	254.96 ± 49.38	204.56 ± 47.11	(22.95,77.84)50.40	1.04(0.44,1.63)	< 0.001*
**RI**					
1st day RI	0.83 ± 0.02	0.87 ± 0.03	0.03 (0.01,0.05)	0.03(0.45,1.64)	< 0.001*
7th day RI	0.85 ± 0.04	0.87 ± 0.03	0.01(-0.01, 0.03)	0.04(-0.3, 0.8)	**0.36**

Data are presented as mean ± SD. *Significant as P value ≤ 0.05. PSV: peak systolic velocity. ESV: End diastolic velocity. RI: Resistive index.MD: mean differences. SMD: standardized mean differences

Peak systolic velocity (PSV) and end-diastolic velocity (EDV) were significantly higher in Group S compared to Group C on both postoperative days 1 and 7 (*p* < 0.001). The resistive index (RI) was significantly lower in Group S (0.83 ± 0.02 vs. 0.87 ± 0.03; *p* < 0.001), indicating improved vascular dynamics.

The incidence of thrombosis was significantly lower in Group S (8%) compared to Group C (36%) (*p* = 0.037). Similarly, functional failure occurred in 32% of Group S versus 64% of Group C (*p* = 0.024). Other adverse events (hematoma, bleeding, infection, ptosis) were not significantly different between groups (Table 4).

**Table 4:** Side effects and failure rate of the studied groups

	Group S (*n* = 25)	Group C (*n* = 25)	*P* value
Side effects			
Hematoma	1 (4%)	2 (8%)	1
Bleeding	2 (8%)	1 (4%)	1
Infection	3 (12%)	1 (4%)	0.609
Ptosis	4 (16%)	0 (0%)	0.109
Failure rate	8 (32%)	16 (64%)	0.024*
Thrombosis	2 (8%)	9 (36%)	0.037*

Data are presented as frequency (%). *Significant as P value ≤ 0.05

## Discussion

This randomized, double-blind trial demonstrates that preemptive stellate ganglion block (SGB) significantly improves AVF hemodynamics and reduces thrombosis and failure rates in hemodialysis (HD) patients undergoing major lower limb orthopedic surgeries. To our knowledge, this is one of the first prospective trials to explore the protective role of SGB on AVFs in this surgical context.

The increase in AVF flow rate in the SGB group is likely due to sympathetic blockade-induced vasodilation. SGB reduces vasoconstrictor tone by inhibiting sympathetic fibers, leading to enhanced arterial blood flow. These findings are consistent with Yildirim et al., who reported significantly elevated PSV in patients receiving preemptive SGB during fistula creation (167.19 ± 31.3 vs. 107.89 ± 15.8 cm/s, *p* < 0.001) [17]. Similar improvements in vascular hemodynamics following SGB have also been reported in other clinical contexts [14, 1].

In our study, improved Doppler parameters (PSV, EDV) and reduced RI further support enhanced perfusion in the SGB group. These physiological improvements may directly contribute to the observed reductions in thrombosis and functional failure, key complications in HD patients with AVFs [6, 9, 10].

A flow rate of ≥ 200 ml/min is generally accepted as sufficient for effective dialysis. In our study, the SGB group maintained average flow rates well above this threshold on both postoperative day 1 and 7, suggesting clinical adequacy. Furthermore, responder analysis showed that 88% of patients in the SGB group-maintained flow > 200 ml/min versus 64% in the control group.

Improved AVF flow rates, as observed in our study, may facilitate better dialysis adequacy by ensuring adequate blood flow for efficient solute clearance. Furthermore, maintaining functional AVFs postoperatively may reduce reliance on temporary central venous catheters, which are associated with higher infection risk and cost. While this study did not directly measure dialysis adequacy or cost, future trials should evaluate these outcomes to better define the broader clinical value of SGB.

In our study, hematoma (4% vs. 8%) and bleeding (8% vs. 4%), infection (12% vs. 4%) and ptosis (16% vs. 0%) were comparable between both groups. SGB group had significantly lower thrombosis incidence (8% vs. 36%) compared with the control one.

Rahimi et al. [1] agreed with our results and stated that SGB groups had significantly lower failure rates compared with the control group. Yildirim et al. [17] showed that hematoma (8% vs. 8%) and bleeding (8% vs. 4%), infection (12% vs. 8%) and ptosis (12% vs. 0%) were insignificantly different between both groups. Also, thrombosis (8% vs. 32%) was significantly SGB group than the control group which agreed with the present findings.

The complication profile was acceptable. Although transient ptosis (indicative of successful SGB) occurred in 16% of Group S patients, no serious adverse events were reported. The use of experienced practitioners and strict adherence to sterile technique under real-time ultrasound guidance contributed to the low complication rate observed, vascular or oesophageal injury, as supported by Bhatia et al. [18] and Soneji & Peng [19].

Performing SGB at the C6 transverse process (TP) level is generally considered safer due to the lower risk of vertebral artery puncture compared to C7 [20]. However, in our study, the block was applied at the C7 level to optimize sympathetic blockade to the upper limb, which may have contributed to the enhanced hemodynamic outcomes. Strict ultrasound guidance and experienced operators were used to mitigate these risks, and no serious complications occurred in our cohort. Nonetheless, this trade-off warrants consideration in clinical practice and future comparative studies. Lastly, anatomical variations and operator experience can affect block success, underscoring the need for standardized training and imaging protocols [21, 22].

We selected the C7 transverse process level for block placement to ensure more comprehensive sympathetic blockade, especially for upper limb vasculature. However, this approach may increase the risk of vertebral artery or pleural puncture compared to the C6 level. Strict ultrasound guidance and experienced operators were used to mitigate these risks, and no serious complications occurred in our cohort. Nonetheless, this trade-off warrants consideration in clinical practice and future comparative studies.

Its limitations include the presence of transient ptosis (Horner’s syndrome), Although the occurrence of ptosis may reveal group allocation, outcome assessments were performed by blinded radiologists using objective Doppler measurements. Future studies may consider photographic documentation or automated Doppler readouts to further minimize observer bias.

The follow-up period in this study was relatively short, limited to 7-day Doppler assessments and 6-week functional AVF use. While sufficient for early postoperative outcomes, longer-term evaluation is essential to determine sustained patency and clinical relevance. Future multicentre trials should incorporate 6- to 12-month follow-up intervals.

Future studies should aim to include larger, multi-centre cohorts and longer follow-up periods to validate and extend these findings. Furthermore, the procedure itself carries inherent risks. Despite the utilization of ultrasound guidance, there remains a risk of block failure due to anatomical variations or identification errors of the necessary landmarks. The dense network of vessels in the head and neck region poses a risk of accidental injection into the wrong site, which could lead to serious complications. This underscores the need for experienced practitioners and precise imaging techniques to minimize such risks. continued efforts to refine the procedure and mitigate associated risks are essential for improving patient outcomes and ensuring the broader applicability of this intervention.

## Conclusions

Pre-emptive ultrasound-guided SGB appears to enhance AVF hemodynamics and reduce thrombosis in HD patients undergoing major surgery. These findings support its potential role as a perioperative vascular preservation strategy, pending confirmation in larger multicentre studies.

## Data Availability

The datasets generated and analyzed during the current study are not publicly available due to institutional restrictions but are available from the corresponding author on reasonable request.
